# Equitable Care for Older Australians: A Comparative Analysis of Aged Care Workforce Shortages in Metropolitan, Rural, and Remote Australia

**DOI:** 10.3390/ijerph22050656

**Published:** 2025-04-22

**Authors:** Nicholas Morris, Susan Jaffer, Stacey Ann Rich, Kate Syme-Lamont, Irene D. Blackberry

**Affiliations:** 1 Care Economy Research Institute, La Trobe University, Wodonga, VIC 3689, Australia; n.morris@latrobe.edu.au (N.M.); k.syme-lamont@latrobe.edu.au (K.S.-L.); 2 Law School, La Trobe University, Melbourne, VIC 3083, Australia; s.jaffer@latrobe.edu.au; 3 John Richards Centre for Rural Ageing Research, La Trobe Rural Health School, La Trobe University, Wodonga, VIC 3689, Australia

**Keywords:** aged care, Australia, rural, workforce, remote

## Abstract

The Australian Royal Commission into Aged Care Quality and Safety has highlighted the chronic shortages of labour to provide care for those aged 65 and over in rural and remote areas of Australia. This descriptive cross-sectional study compares the availability of care provision in metropolitan regions with that in rural and remote regions. We analysed the 2021 Australian Census, grouped according to Aged-Care-Planning Region (ACPR), and investigated the numbers of people aged 65 years and over with different levels of care need, both in residential care and in-home. The available workforce in each ACPR was also examined in detail, using occupational classifications reported in the Census, and shortages of doctors, nurses, allied health and other care workers were identified. Overall, an additional 492,416 care hours were needed per week (or 12,958 full-time equivalent (FTE) care workers) in order to bring remote community ACPRs to parity with provision in metropolitan ACPRs. A further 95,342 FTE workers were needed in rural ACPRs to bring these areas to parity with metropolitan ACPRs. Our findings underscore the ongoing disparities in aged care workforce availability between metropolitan, rural, and remote regions of Australia. Addressing these workforce shortages is crucial to ensuring equitable access to care for older Australians, regardless of their geographical location. The implementation of targeted strategies to enhance workforce recruitment, retention, and training in these underserved areas is essential to bridge the gap and improve the quality of care provided to older adults in rural and remote communities. Such strategies could include targeted recruitment campaigns and incentives for professionals to relocate; further capacity for clinical placements and supervision in rural areas; tailoring funding and employment models for rural needs; and strengthening vocational education in regional areas.

## 1. Introduction

As the Australian population ages, and the migration of younger people to the metropolitan areas of Australia accelerates, shortages of labour to provide care for those 65 years and over in rural and remote areas are increasing. The Australian Royal Commission into Aged Care Quality and Safety found ongoing workforce recruitment and retention challenges particularly outside metropolitan areas and highlighted skill shortages for registered nurses, personal care workers, and home care in remote areas [1]. This problem has also been highlighted in recent reports by the Committee for Economic Development of Australia (CEDA), which has estimated that the aged care workforce is now facing an annual shortfall of up to 35,000 staff, with 8,000 additional workers needed to meet international best practice standards [2]. Following the Royal Commission, the Australian Government is seeking to improve aged care workforce availability through wage increases, training and education opportunities, and support providers to improve skills and work culture [3].

However, the care and support needs of older adults go beyond that of aged care workers. The Australian Institute of Health and Welfare Older Australians report [4] tracks older adults’ usage of health services that extend beyond aged care to include primary care, allied health, mental health, dental, pharmaceutical, and palliative services. Older adults rely more heavily on sectors of the broader health workforce than those under the age of 65 years. For example, allied health services are accessed at a much greater rate in the 65 years and over age group than the under 65 years (65% vs. 32%) age group, and pharmaceutical-dispensing rates are highest in the 85 and over age group [4], suggesting that these professions provide an integral component of care for older adults. Workforce shortages are evident across these and many other sectors that are required for the care needs of older adults both in Australia and worldwide [5].

Recent investigation of current, emerging, and future skills requirements in Australia indicates high demand for many of the broader professional groups required to care for and support older adults [5]. Care workers’ wages in Australia are primarily determined by awards set by the Fair Work Commission, and it is hoped that the recent increase in the Aged Care Award, the Social, Community, Home Care, and Disability Services (SCHADS) and Nurses awards will attract more workers to the industry.

**Figure 1:**
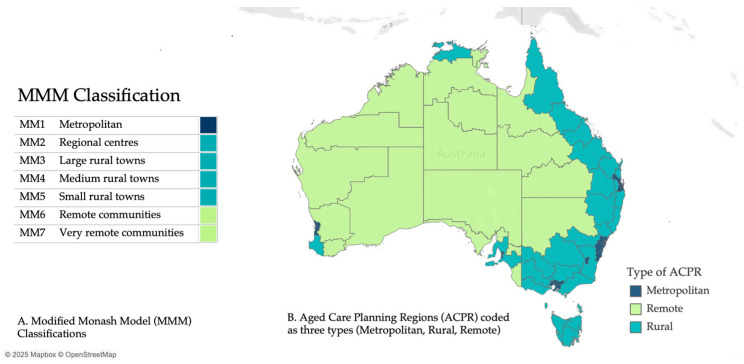
Modified Monash Model (MMM) classifications [8].

However, some of the highest vacancy rates for health and care occupations are seen in rural areas with the lowest number of healthcare workers (including General Practitioners, nurses, and allied health workers) per capita being evident in small rural towns (MMM5; see Figure 1) [6]. With the burden of disease, all-cause death rates per 100,000 people, and the likelihood of death from potentially avoidable causes increasing as remoteness ranking increases [7], the disparity between metropolitan and rural Australia is evident, and this is exacerbated by the distances that care workers need to travel. Nonetheless, the extent of geographic disparities in the workforce required to support older adults in Australia is unknown.

Our previous article [9] used detailed data on the availability of residential and in-home care to highlight gaps in care provision for rural and remote communities relative to that enjoyed by those living in metropolitan areas. This article extends that analysis to explore care needs, and the availability of care workers, on a geographical basis.

The current article poses the question, can the existing formal workforce meet the care needs of older adults aged 65 years and over in rural and remote regions of Australia? The formal workforce is defined here as paid care services provided by professionals employed under six categories associated with the provision of care for older adults, being allied health professionals and assistants, personal support and care workers, nurses, doctors, dentists and oral health professionals, and administration and management professionals (refer Appendix C). The formal care workforce does not include the informal care provided by family, close relatives, friends, and neighbours [10].

Aged-Care-Planning Regions (ACPRs) in Australia are geographic areas used by the government to plan and allocate aged care services. These 73 regions are based on Statistical Area Level 2 (SA2) boundaries. As in our previous article [9], this analysis compares the availability of care provision in metropolitan regions with that in rural and remote ACPRs.

Labour force shortages in rural and remote ACPRs are estimated by comparing available care hours, per person needing care, with those in metropolitan ACPRs. The severity of care need is considered by utilising data on long-term health conditions and the need for assistance with core activities. It is hypothesised that rural and remote ACPRs have lower levels of care workforce per capita compared to metropolitan ACPRs.

## 2. Objectives

The objectives of this study are multifaceted and aim to address critical aspects of aged care services in Australia. Firstly, it seeks to evaluate disparities in the availability of the aged care workforce across Australian ACPRs. This involves identifying variations in workforce distribution and understanding how these disparities impact the delivery of care. Secondly, the study aims to quantify the care needs of individuals and the corresponding care hours available to meet these needs. This includes assessing the adequacy of care provided and identifying gaps in service delivery. Lastly, the study endeavours to estimate workforce shortages by region type, distinguishing between metropolitan, rural, and remote areas. By doing so, it aims to highlight specific challenges faced by different regions and propose targeted solutions to address these workforce shortages effectively.

## 3. Materials and Methods

This is a descriptive cross-sectional study using secondary data from the 2021 Australian Census. Our analysis enables the population aged 65 years and over to be grouped based on ACPRs into different categories of care need and permits identification of those who are cared for in a residential setting versus those who are cared for in their own home. We compared the care needs per ACPR, measured in terms of hours per week, with the hours worked by the available workforce. The workforce was then examined in some detail, using occupational classifications reported in the Census, and relative shortages of doctors, nurses, allied health, and other care workers were identified. For the purposes of the analyses, care work was divided into direct care, such as that provided by trained care professionals, and indirect care, such as that which provides the preconditions for care (for example, cleaning, cooking, and maintenance).

Ethics approval, consent to participate, and permission to access data were not required since data were de-identified and publicly available. In our use of the Census, we relied on the privacy-protection processes developed by the Australian Bureau of Statistics, through TableBuilder Pro, to ensure that individual records could not be identified.

### 3.1. Data Sources and Extraction

The primary source of data was the full 2021 Census, which contained the responses of 25,370,037 individuals across Australia and which included information on health status, employment, and provision of informal care. The Census was accessed between November 2023 and December 2024 using ABS TableBuilder [11]. The Census identified where respondents resided on Census night (10 August 2021), including by ACPR, as well as providing a variety of health indicators. The indicators utilised in particular were as follows: whether respondents report a diagnosis of one or more long-term health conditions, whether they have a need for assistance with day-to-day activities, and whether they are living in their own home or in a residential facility. The analysis focused on those aged 65 and over and younger adults requiring care due to complex health conditions or disabilities that demand specialised support (for example, someone with a severe spinal cord injury might need residential care to access round-the-clock assistance, rehabilitation services, and tailored accommodation).

The Census also included extensive and detailed analysis as to the occupation and industry of individuals living in each ACPR. The most detailed data available have been used to identify those who work in the care industry and group these by area of expertise. Hours worked were also reported, and this information has been used to estimate the number of care hours provided by each category of care worker. In October 2023, the Australian government imposed mandatory requirements for the provision of care minutes, at different levels of care need, from nurses and personal care workers in residential care settings. These requirements have been used to estimate the care hours required for residents of care facilities with different levels of care requirements. Appendix A summarises the new mandatory requirements that have been introduced.

The two strands of analysis were drawn together to explore care needs and provision of paid care hours. This was used to highlight gaps in the formal labour force and show how this varied between ACPRs. Individuals aged ≥65 years recorded in the 2021 Census, classified by ACPR, were included in the analysis of need, while individuals with missing age or location data, or those living overseas on Census night, were excluded.

### 3.2. Data Analysis

Descriptive statistics were used to summarise workforce availability and care needs. Rates per 1000 population aged ≥65 were calculated. No inferential tests were applied due to the use of complete Census data.

The ACPR were classified by geographical remoteness using the Modified Monash Model (MMM) scale [12], as depicted in Figure 1. 

The MMM is a key tool being used increasingly by the Australian Commonwealth Department of Health to “describe geographical access” [13]. In 2016, it was introduced into, for example, the Australian Longitudinal Study on Women’s Health (ALSWH) as a measure of remoteness [14].

Appendix B provides a table listing the Aged-Care-Planning Regions by state and reports a simple average of the MMM scores for the Statistical Area 2 (SA2) areas which comprise them. The average MMM scores for each ACPR were then grouped into three categories: metropolitan (MMM 1.99 and below), rural (MMM 2 up to 5), and remote communities (MMM 5 and over). This study attempted to identify the workforce currently available in each ACPR to provide care specifically for older people. This was conducted using the OCCP (occupation) field at the 6-digit level to identify direct care workers and the detailed INDP (industry) field for aged care residential services to identify indirect care workers. Appendix C summarises the occupations that have been selected as relevant to aged care. A total of 1,214,160 workers across Australia are in this category, including managers, receptionists, nurses, care workers, doctors, specialists, and allied health. A further 68,770 indirect workers work in aged-care residential services, including cleaners, cooks, gardeners, and maintenance workers.

Individuals in the Census aged 65 years and over have been grouped according to their aged care situation (TableBuilder code RNLP: residential status in a non-private dwelling), as guest, patient, inmate, or other resident by their need for assistance with core activities (TableBuilder code ASSNP: core activity need for assistance) and by the number of long-term health conditions they have (TableBuilder code CLTHP: count of selected long-term health conditions).

Two strands of analysis were pursued. The first examined differences between metropolitan regions on the one hand, and rural and remote regions on the other, in terms of available workforce compared to need. Thus, the numbers of workers in each occupation group and their average hours were compared to the population aged 65 years and over in the three region types: metropolitan, rural, and remote.

The second strand of analysis used Census data on the care needs of individuals in the different ACPRs in terms of whether they required assistance with daily activities and the extent of any chronic health conditions. Individuals were grouped into four care need categories to which required care minutes were applied based on the new government requirements. Although government standards apply only to residential care, similar care levels were assumed for individuals with comparable health needs at home based on principles of equity in service provision. The total required care hours were then compared to total available care hours from the nursing and personal care workforce.

## 4. Results

### 4.1. Population Aged 65 and over

Table 1 shows the breakdown of the Australian population according to the ACPR categories. While there were fewer people aged 65 years and over living in rural and remote areas compared to those living in metropolitan ACPRs, the proportion of people aged 65 and over is higher in both cases than that in metropolitan areas.

**Figure 2:**
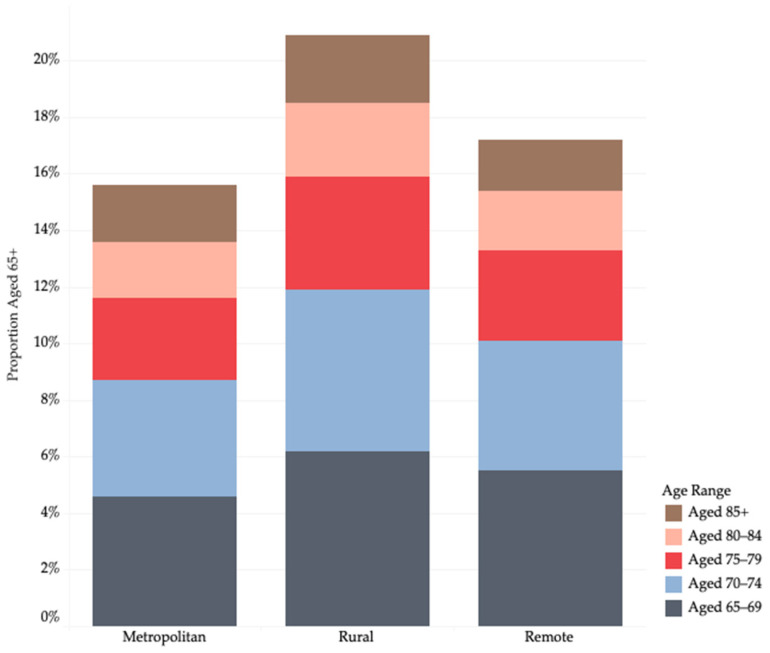
Proportion of population aged 65 years and over (in ranges) in each ACPR type. Source: Analysis of 2021 Census, using ABS TableBuilder Pro, accessed between 16 November 2023 and 18 December 2024.

Figure 2 provides further detail, showing that 5% of the population in rural ACPRs are aged 80 or over, compared to 4.1% in metropolitan regions and 3.9% in remote communities. This is important because people aged 80 and over typically require more care [4].

**Figure 3:**
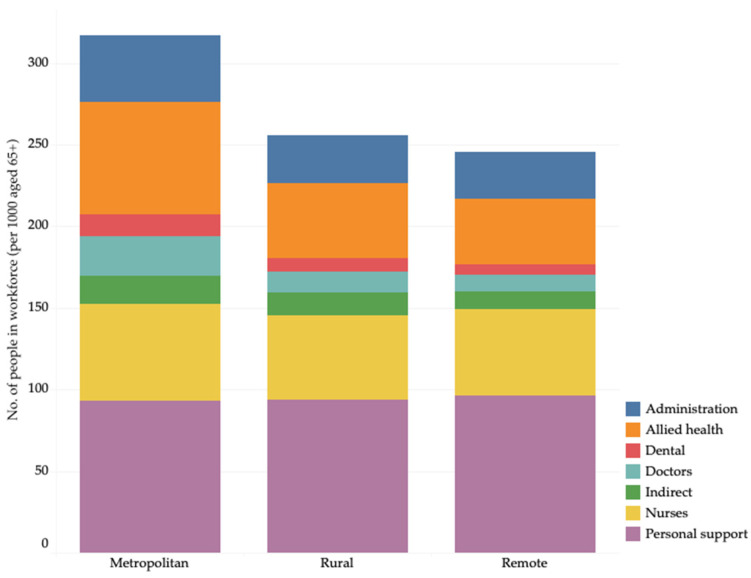
Rate (per 1000 aged 65 years and over) of aged care workforce by type of ACPR. Source: Analysis of 2021 Census, using ABS TableBuilder Pro, accessed between 16 November 2023 and 18 December 2024.

Analysis of the Census data showed there were proportionately fewer workers available in rural and remote ACPRs to serve these older populations. Figure 3 shows that whereas there were 317 relevant industry workers per 1000 people aged 65 years and over in metropolitan areas, there were only 256 in the rural regions and just 245 workers per 1000 people in remote communities. The details of the age distribution in rural and remote ACPRs are provided in Appendix D.

### 4.2. Care Needs

Age distribution influences the care needs in each ACPR, whether in residential settings or at home. Appendix E, Table A3 shows that there were 208,892 individuals identified by the 2021 Census as living in residential care facilities (some 7.7% of the 65 and over population), of whom 71,665 (4.7% of the 65s and over in those regions) were in rural ACPRs and 6674 (4.5% of the 65s and over) in remote ACPRs. A higher proportion of those in residential care in remote communities had the highest care needs (28.2% compared to 23% in metropolitan ACPRs).

Appendix E, Table A4 shows that there were 859,609 people (17.1% of the population aged 65 and over) who required assistance but still lived at home, of which 287,262 were in rural ACPRs and 25,754 were in remote communities. The remaining population aged 65 and over, and living at home, required some care (from doctors, dentists, nurses, etc.) but less intensely.

### 4.3. Care Workforce

Caring both for those in residential care and those with care needs at home requires a substantial workforce. Appendix F, Table A5 shows how the number of care workers varies across types of ACPR. In aggregate, these figures are broadly consistent with the 2020 aged care workforce Census, which found—for Australia as a whole—277,761 nurses and carers in residential aged care, 80,340 in the home care packages program, and 76,096 in the Commonwealth Home Support Program, a total of 434,197 [15]. The Census also records hours worked per week, which allowed the total care hours provided by each category of care workers to be estimated (the available data provide this information in ranges, and we have taken the mid-point of each range to estimate total hours). The results, shown in Appendix F, Table A6, estimate 40.35 million care hours per week provided across Australia, or 6.79 h per person aged 65 and over.

### 4.4. Shortfall in Available Care Hours

Table 2 brings the analysis of care workers together and estimates how many additional workers of each type are needed to bring the available hours in remote ACPR regions to the same level, per person aged 65 and over, as in metropolitan ACPRs. This equates to an additional 15,656 workers in remote regions, assuming they work the same hours per week as existing workers. In full-time equivalent terms, for a 38 h week, this amounts to an additional 12,958 full-time equivalents (FTEs) in remote communities.

Table 2 also shows equivalent results for rural ACPRs (those with MMM scores between 2 and 5). Here, an additional 110,361 workers are needed, across all categories, if they worked, on average, the same number of hours per week as existing workers. This translates to 95,342 FTEs workers on the basis of a 38 h week.

**Figure 4:**
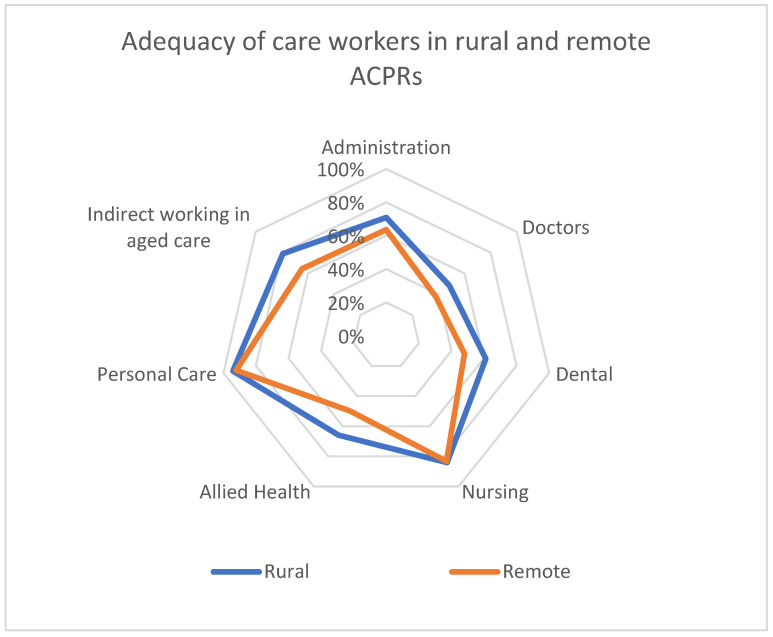
Adequacy of care workers in rural and remote ACPRs. Note: Achieving 100% in these diagrams would mean that care provision was equivalent to that in metropolitan ACPRs.

Figure 4 below shows the shortfall implied for workforce hours provided by administrators, doctors, dental professionals, nurses, allied health, personal care, and indirect workers for rural and remote regions, relative to metropolitan regions. The figure compares the shortages of care workers of different types between rural ACPRs and remote community ACPRs, relative to care workers in metropolitan ACPRs. In both rural and remote ACPRs, there is a particular shortage of doctors.

Table 3 sets out the empirical findings according to type of ACPR by the category of care need. The table also estimates the care hour requirements of those receiving care at home on the assumption that the same number of care minutes are required for those with the same care needs.

Overall, a total of 23.7 million hours is required per week, from nurses and personal care workers. The provision of care in an in-home setting is not yet a mandatory requirement; nonetheless, it is reasonable to assume that people with similar needs in terms of assistance with core activities and complex health conditions need similar care, whether in a residential or in-home setting.

Table 4 compares the hours per week required to provide the government-mandated level of care in both residential and in-home settings with the care hours identified as available above.

Table 4 reveals that, overall, some 5.7 million extra care hours per week are required from nurses and personal care workers to meet the standards required for residential care and implied for in-home care.

**Figure 5:**
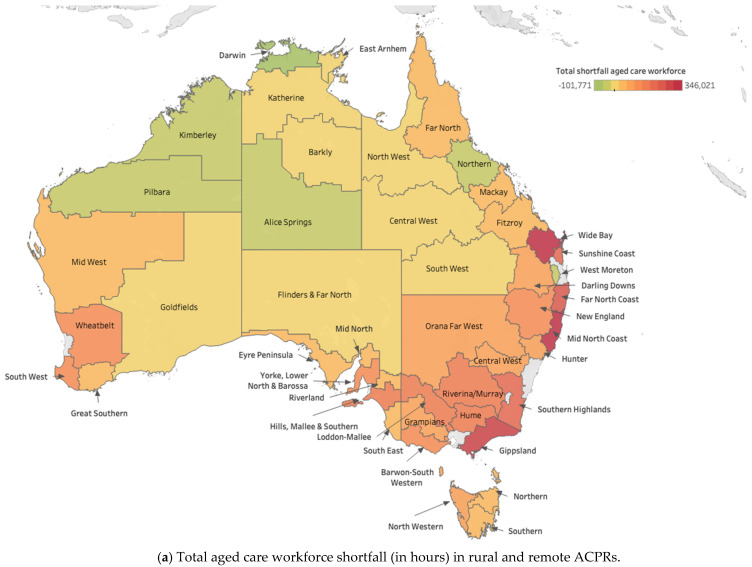

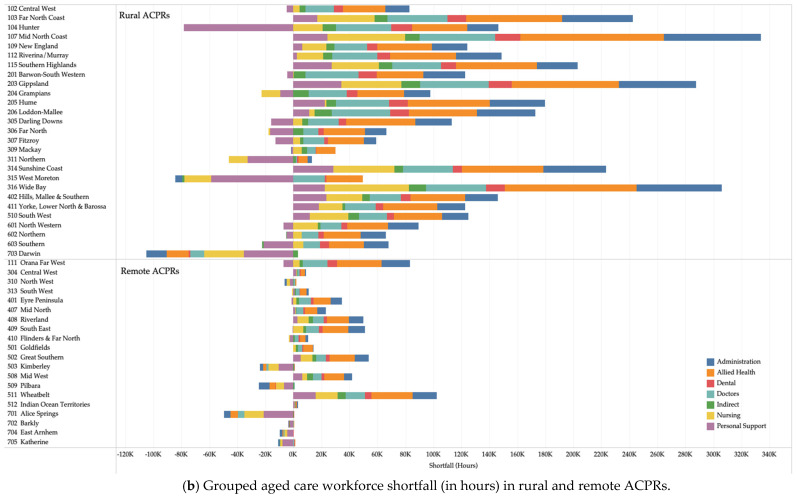
Aged care workforce shortfall in rural and remote ACPRs.

The shortfall in availability of care workers varies by ACPR, partly because the population age profile varies. This means that detailed investigation of suitable local solutions is warranted at the level of individual ACPRs. Figure 5 below explores, for rural and remote ACPRs, where additional workers are needed, and of what type. The classifications of care workers used are described in Appendix C.

Given the vast geographical area of Australia, variations exist within rural or remote communities as a result of population profile, local industry, access to transportation, and socioeconomic status. For example, while shortages in remote ACPRs are common, these are less severe in the mining communities where the age profile is younger. Meanwhile, there are particularly large shortfalls in the Wheatbelt, Mid-West, Great Southern, Riverland, Southeast, Central West Queensland, and Orana Far West ACPRs.

Nationally, however, the ACPRs with the most acute shortfalls were rural ACPRs that flank the more densely populated centres along the East Coast. All ACPRs with an estimated total shortfall of over 150,000 h in comparison to metropolitan areas were in these three coastal states: Wide Bay (>305k h), Sunshine Coast (>223k h) in Queensland; the Far North Coast (>242k h), Mid North Coast (>333k h) in New South Wales, Southern Highlands (>203k h), Gippsland (>287k h), Hume (>179,000 h), and Loddon-Mallee (>172,000k h) in Victoria.

While it is immediately obvious that a shortage of general practitioners and allied care professionals in rural areas is problematic, it is less obvious why thought should be given to the administration workforce attached to care provision. A lack of administrative support often results in care workers incorporating up to 2 h of administrative work into their day, reducing time available for patient care. Further, this administrative burden is associated with higher likelihood of intention to leave aged care professions [16].

Figure 5b above shows that there is a shortage of many categories of care professionals in most rural and remote ACPRs compared to their metropolitan equivalents. There appears to be a particular shortage of doctors and allied health professionals. In the case of doctors, and some specialists, it is possible that those needing care could travel to nearby metropolitan ACPRs to see them. However, transport difficulties from peri-urban areas to metropolitan centres in Australia arise from limited public transport options, poor road infrastructure, high costs of private vehicle ownership and maintenance, and restricted affordable public transport choices, and the distances involved can be quite substantial.

## 5. Discussion

This article extends the existing knowledge of aged care inequity experienced by older people in rural and remote Australia. Our findings highlight the presence of aged care workforce shortages across Australia, but particularly in rural and remote community ACPRs.

Similar studies have been carried out internationally, utilising secondary data to highlight critical gaps in health workforce distribution and their implications for equitable health care. For instance, Wang et al. [17] analysed workforce data to uncover disparities in distribution across economic regions in China, emphasising equity as a health care goal. Garg et al. [18] highlighted the essential role of Human Resources for Health (HRH) in achieving universal coverage, showing how Health Labour Market Analysis (HLMA) can reveal gaps in workforce policies, including geographic distribution. Cortie [6] identified factors associated with shortfalls in the healthcare workforce MMM regions, providing insights into geographic disparities.

While these studies underscore the need for a focused analysis of the distribution of health workforce, they do not directly address the full range of services required by older adults located in the geographical areas used to plan ACPR. The analysis in this article demonstrates that the availability of aged care workers both in residential care and in-home care in rural and remote ACPRs in Australia was lower per person aged 65 and over than in metropolitan settings. The shortage was most acute in rural ACPRs. These findings are consistent with research in Canada, where Ariste found that there were three times as many physicians per 1000 older adults in urban areas than there were in rural Canada (18.3 and 6.0, respectively) [19].

In response to the Royal Commission into Aged Care Quality and Safety [1], the Australian government has been seeking ways to improve workforce shortages and is developing new funding models and quality standards. These include the introduction of mandatory care minutes, as presented in Appendix A. Since July 2023, residential aged care homes across Australia were required to have a registered nurse on-site and on duty 24 h a day, 7 days a week, unless granted a 12-month exemption. Additionally, residential aged care homes are required to deliver at least 215 care minutes per resident per day, including 44 min with a registered nurse. The latest Government report [20] indicated that between April and June 2024, the average care minutes provided were 207.71 with 41.44 care minutes by registered nurses. However, only just over half of services were meeting either the total care minutes or care minutes by registered nurses, and only 45.54% of services met both criteria. The report found that the average care minutes in MMM 5–7 were higher than other MMM areas, which may be due to the exemptions being granted and additional funding for remote areas. Our analysis has identified the significantly greater shortfall in available care hours that follows if older adults living at home required similar standards of care to those in residential aged care facilities.

The Aged Care Royal Commission [1] has also highlighted problems with the quality-of-care provision, including in metropolitan areas. The chronic workforce shortage has a direct impact on the quality of care delivered in residential aged care settings. Data from 25 residential aged care homes in Australia in 2021 found that only one half of evidence-based quality indicators were met [21]. The areas of particular need included skin integrity, end-of-life care, infection, sleep, medication, and depression. Furthermore, older Australians face an average 90 days waiting period to access home care packages. This is despite the fact that the provision of home care packages is acknowledged to be beneficial in terms of reducing premature mortality and admissions to aged care. [22].

The Australian population is dispersed, the lack of transport makes service provision difficult, and older people often cannot travel to access aged and healthcare [8]. In remote communities, the shortage of aged care workers is exacerbated by the extra travel time these workers need to travel (sometime hundreds of kilometres). Travel might affect the availability of care hours, allowing extra time to be taken up in additional travel, for those categories of care workers who might be expected to travel including for agency workers. Some allowance has been made in our analysis, but if there are additional travel time and costs, there may be further substantial shortfall, similar across the three types of ACPR, contributing to the need for informal care to be provided by family, friends, and the wider community. As illustrated in Figure 5a,b, while the workforce shortfall in rural ACPRs should also be addressed, detailed investigation of how to address the problem in specific remote locations is warranted.

Careful analysis of the data presented in Table 4 above reveals that rural and remote ACPRs, despite having less nurses and far less other health and allied health professionals, can have more lower-paid carers, which is why the overall shortage is similar across the three types of ACPR. These findings directly address the study objectives by quantifying regional disparities in workforce supply and confirming the hypothesis of significant inequality between ACPRs. They thus provide compelling evidence for reform.

The Aged Care Royal Commission highlighted how the quality of care provided to older people in Australia was woefully inadequate, largely due to a shortage of appropriately trained staff. This article shows that this situation is worse in rural and remote ACPRs than it is in metropolitan settings. The solutions are obvious: enhancing retention, improving recruitment, encouraging return to practice, and drawing on international human resources [23,24,25]. All of this requires, of course, adequate funding and rates of pay that enable the workforce to flourish in more remote regions. As shown in Appendix C, the remote communities vary widely in the proportion of people aged 65 and over. The analysis finds that the shortfall of aged care workers likewise varies between individual remote ACPRs. In some of the remote ACPRs—particularly those with large mining activities—the number of health professionals available to care for those aged 65 and over is more adequate.

We observed stronger demand for aged care workers in rural regions closer to metropolitan ACPRs. This may be due to the higher proportion of older population in the rural communities. However, it has been common, even prior to the COVID-19 pandemic, for populations to migrate from metropolitan areas to the surrounding towns and coastal areas that offer greater lifestyle and affordability [26]. However, without adequate infrastructure for local services and public transport, inequitable access to care may worsen as often these new towns are outside of the metropolitan service catchment, and the existing care services are unable to meet the demand of the influx of older people requiring care.

The Australian Government has recently implemented several policies to address workforce shortages in the aged care sector. These include expedited visa processing for aged care workers to attract international professionals; streamlined services through the Support at Home Program to enhance efficiency and job satisfaction; significant funding for pay rises to retain skilled nurses; strengthened education programs and regional incentives to build a skilled workforce; and mandatory workforce planning by providers to ensure quality care and address shortages. However, the Inspector General of Aged Care stresses that there remains an urgent need “boost numbers in regional, rural and remote areas now” [27]. One specific recommendation is to review the adequacy of the non-metropolitan Base Care Tariff.

However, it is not clear that the changes so far envisaged will be sufficient to address the workforce imbalances between rural/remote care and its metropolitan equivalent. Further attention needs to be given to targeted recruitment campaigns and incentives for professionals to relocate; further capacity for clinical placements and supervision in rural areas; tailoring funding and employment models for rural needs; and strengthening vocational education in regional areas. The policies suggested in the recently published Regional, Rural, and Remote Jobs and Skills Roadmap [28] should be actively explored.

While there is no single and short-term solution to address issues of this magnitude, the growing uptake of telehealth, virtual aged care nursing, and technology may ease the delivery of skilled aged care provision. The Australian Government report, Aged Care Data and Digital Strategy 2024–2029 [29] included the launch of a virtual residential aged care nursing to alleviate care minutes requirement and the use of Artificial Intelligence (AI). However, as the majority of aged care services from residential aged care to home care packages require local staff and face-to-face delivery, a multi-pronged approach is required beyond funding alone to be sustainable. Aligned with older people’s preference for ageing in place, our research shows the importance of the whole of the community to support their older population [30]. This may involve deploying local community members as volunteers to support older people and their caregivers to complement formal aged care provision.

Family members and friends, as well as the wider community, are at present, of necessity, filling the gaps left by an inadequate formal workforce, and this situation will continue for the foreseeable future. Despite their devotion to those they care for, they often do not have the skills of a properly trained nurse or care worker. Additionally, they certainly cannot fill the gaps left by an inadequate number of GPs, specialists, and allied health professionals. The need to provide such large amounts of informal care will also place enormous strain on these carers, affecting family life and their other activities. Considerably more attention needs to be given to the role of informal care and what can be done to support caregivers.

This article has several strengths and some limitations. We performed data linkage and examined routinely collected (de-identified) national Census data, which provides data that are rich and comprehensive. There are, however, assumptions and estimations required for our analyses, which limits the scope of our article. Census data may lack the precision and depth required to fully capture the nuanced health and care needs of individuals, potentially leading to an incomplete understanding of the workforce and service requirements. Second, the study assumes that care needs at home are equivalent to those in residential settings. This assumption may oversimplify the complexities of home-based care, where factors such as family support, accessibility, and individualised arrangements can significantly alter care requirements.

COVID-19 had a severe impact on the aged care sector and on its workforce. This article relies on data collected in August 2021, a time when Australia was still pursuing a zero-COVID strategy, many cities were in lockdown, and the first wave of vaccinations was being implemented. The impact of COVID-19 upon workforce shortages, workplace demands, worker wellbeing, and intentions to quit the aged care profession has previously been documented [31].

After public consultation, the Aged Care Bill 2024 was passed by Parliament on the 25 November 2024 and will become the new Aged Care Act from the 1 July 2025. The new Act will “clearly set out the obligations of aged care providers and legislate requirements that protect the rights of older people in Australia to safe, quality care” [32]. Despite improvement in care minutes, migration policy change, increases in minimum salaries of aged care workers, aged care funding model review, and investment in aged care technology infrastructure, the implementation of Aged Care Royal Commission recommendations is still far from complete [27]. Demand for in-home care in the future will continue to escalate as older people prefer to age in place.

## 6. Conclusions

A third of older Australians live in rural and remote communities [24], a higher proportion than in metropolitan areas. This article demonstrates that there is currently a shortfall of aged care workers in rural and remote ACPRs. This highlights the urgent need for tailored policy interventions to guarantee fair and adequate care for older Australians across all regions.

## Figures and Tables

**Table 1:** Population by type of ACPR.

Aged-Care-Planning Region	Average MMM Classification	Average MMM	Population	Population Aged 65 and over	Proportion Aged 65 and over
Metropolitan	1.99 and below	1.33	17,265,810	2,711,626	15.7%
Rural	2 up to 5	4.19	7,241,860	1,511,126	20.9%
Remote	5 and over	6.02	862,367	148,635	17.2%

Source: Analysis of 2021 Census, using ABS TableBuilder Pro, accessed between 16 November 2023 and 18 December 2024. Source: Analysis of 2021 Census, using ABS TableBuilder Pro, accessed between 16 November 2023 and 18 December 2024.

**Table 2:** Additional care workforce required to bring rural and remote ACPRs to parity with metropolitan ACPRs (this is the number of hours that would have been required in 2021, the year the data were collected).

	Additional Hours for Parity		Additional Workers Required (at Average Hours)		Additional FTE Workers Needed *	
	Remote	Rural	Remote	Rural	Remote	Rural
Direct care						
Administration	94,146	709,369	2392	18,024	2478	18,668
Doctors	86,072	697,952	2414	19,574	2265	18,367
Dental	27,947	211,219	1043	7882	735	5558
Nursing	52,338	461,094	1668	14,696	1377	12,134
Allied health	168,774	1,129,488	5367	35,919	4441	29,723
Personal care	35,785	249,737	1264	8820	942	6572
Indirect care						
Indirect workers in aged care	27,354	164,136	908	5446	720	4319
Total direct and indirect	492,416	3,622,995	15,656	110,361	12,958	95,342

* Based on a 38-h week, see https://employsure.com.au/blog/understanding-part-time-vs-full-time-employment-in-australia-a-comprehensive-guide. Source: Analysis of 2021 Census, using ABS TableBuilder Pro, accessed between 16 November 2023 and 18 December 2024.

**Table 3:** Daily care minutes * required, and implied hours per week, by type of ACPR.

Aged-Care-Planning Region Type	Metropolitan	Rural	Remote	Total	Implied Weekly Hours
Residential daily care minutes based on government mandatory requirements					
Highest care: Assistance and 3 or more conditions	8,257,562	5,063,966	519,318	13,840,845	1,614,765
High care: Assistance and 2 or less conditions	11,588,585	6,331,821	556,792	18,477,197	2,155,673
Medium care: LT conditions but no assistance	2,727,582	1,401,829	118,404	4,247,815	495,578
Low care: No LT conditions	1,709,290	817,520	89,430	2,616,240	305,228
Aged 20–64, inmates, need assistance	1,849,156	1,011,912	82,926	99,259	11,580
All in residential care	26,132,175	14,627,047	1,366,869	39,281,356	4,582,825
In-home daily care minutes based on government mandatory requirements					
Requires highest care: Assistance and 3 or more conditions	31,940,644	18,221,295	1,537,015	51,698,953	6,031,544
Requires high care: Assistance and 1 or 2 LT conditions	40,999,790	18,305,486	1,616,593	60,921,869	7,107,551
Requires medium care: Assistance, no LTC and no Assistance, 3 or more LTC	31,605,145	18,235,789	1,702,987	51,543,921	6,013,457
In-home, requiring care	104,545,578	54,762,569	4,856,595	164,164,743	19,152,553

* Care minutes are the direct care time delivered to residents by registered nurses, enrolled nurses and personal care workers (including nursing assistants). Long term (LT). Note: As discussed in Section 3.1, the government mandatory requirements for nurses and personal care workers in residential care were set in October 2023. Source: Analysis of 2021 Census, using ABS TableBuilder Pro, accessed between 16 November 2023 and 18 December 2024.

**Table 4:** Shortfall in available hours from nurses and accredited care workers by type of ACPR.

Aged-Care-Planning Region Type	Metropolitan	Rural	Remote	Total
Residential and in-home hours required	15,245,738	8,905,001	871,285	25,022,023
Nurse hours available	5,065,509	2,437,119	260,877	7,763,504
Care worker hours available	7,113,767	4,018,180	421,534	11,553,480
Shortfall in available hours	3,066,462	2,449,702	188,874	5,705,039
Shortfall as proportion of hours required	20.1%	27.5%	21.7%	22.8%
Extra travel time		10%	20%	

Source: Analysis of 2021 Census, using ABS TableBuilder Pro, accessed between 16 November 2023 and 18 December 2024.

**Figure A1:**
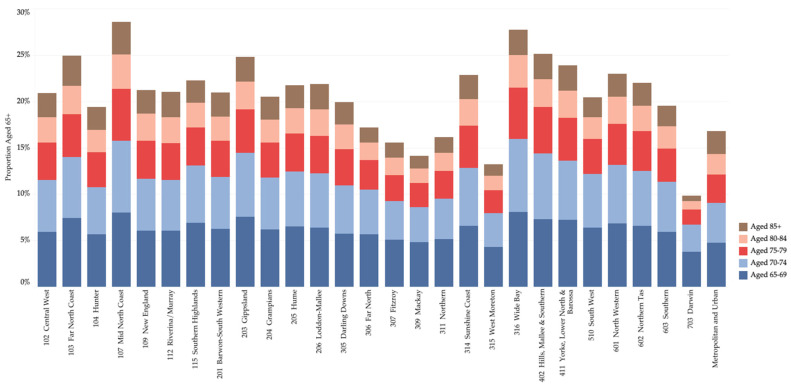
Proportion of population aged 65 years and over in individual rural ACPRs, compared to metropolitan total.

**Figure A2:**
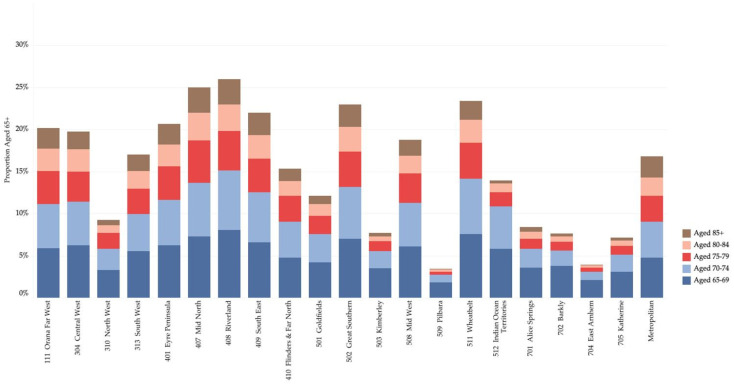
Proportion of population aged 65 years and over in individual remote ACPRs, compared to metropolitan total.

## Data Availability

The data extracted and analysed for this article was obtained from ABS TableBuilder Pro, with permission from the Australian Bureau of Statistics, https://tablebuilder.abs.gov.au/webapi/jsf/login.xhtml. The specific dataset used was the 2021 Census-counting persons, place of usual residence, see https://www.abs.gov.au/statistics/microdata-tablebuilder/available-microdata-tablebuilder/census-population-and-housing#data-downloads. Data was accessed between 16 November 2023 and 18 December 2024.

## Appendix A. Mandatory Care Minutes in Residential Aged Care

The Australian government, partly in response to the criticisms levied by the recent Royal Commission [1], has issued mandatory care minute requirements for those in residential care. These care minutes must be provided by registered nurses (RNs), enrolled nurses (ENs), or personal care workers and assistants in nursing (PCWs/AINs). Direct care activities may include both direct in person assistance and those that are not face to face (for example, writing up care plans or organising a referral for an allied health service are considered direct care activities that are not carried out face to face).

**Table A1:** Mandatory care minutes for residential care.

Care Recipient Classification	Combined Staff Daily Amount (Minutes)	Registered Nurse Daily Amount (Minutes)	Definition
Class 1	317	57	Admit for palliative care
Class 2	110	30	Independent without compounding factors
Class 3	143	32	Independent with compounding factors
Class 4	115	28	Assisted mobility, high cognition, and without compounding factors
Class 5	157	39	Assisted mobility, high cognition, and with compounding factors
Class 6	152	34	Assisted mobility and moderate cognition
Class 7	186	36	Assisted mobility and low cognition
Class 8	200	38	Non-mobile and high cognition
Class 9	202	46	Non-mobile and moderate cognition
Class 10	282	56	Non-mobile and low cognition
Class 11	274	41	Non-mobile, high function, and pressure sore risk
Class 12	269	42	Non-mobile, moderate function, and pressure sore risk
Class 13	317	57	Non-mobile, low function, and pressure sore risk

These took effect on 1 October 2023 and are set to increase on 1 October 2024. We used the 2023 level and related these categories to the level of care need outlined above for both those in residential care and in-home care.

## Appendix B. Aged-Care-Planning Regions and MMM Classification

**Table A2:** Aged-Care-Planning Regions (ACPRs) and Modified Monash Model (MMM) indicator of remoteness.

State/Territory	ACPR Code	ACPR Name	Average MMM Classification
New South Wales	101	Central Coast	1.75
	102	Central West	4.98
	103	Far North Coast	3.83
	104	Hunter	2.20
	105	Illawarra	1.96
	106	Inner West	1.00
	107	Mid North Coast	4.33
	108	Nepean	1.63
	109	New England	4.93
	110	Northern Sydney	1.21
	111	Orana Far West	5.36
	112	Riverina/Murray	4.69
	113	South East Sydney	1.21
	114	South West Sydney	1.62
	115	Southern Highlands	4.55
	116	Western Sydney	1.18
Victoria	201	Barwon—South Western	4.14
	202	Eastern Metro	1.20
	203	Gippsland	4.73
	204	Grampians	4.60
	205	Hume	4.65
	206	Loddon-Mallee	4.62
	207	Northern Metro	1.16
	208	Southern Metro	1.24
	209	Western Metro	1.08
Queensland	301	Brisbane North	1.00
	302	Brisbane South	1.36
	303	Cabool	1.62
	304	Central West	7.00
	305	Darling Downs	4.90
	306	Far North	4.65
	307	Fitzroy	4.84
	308	Logan River Valley	1.93
	309	Mackay	4.96
	310	North West	6.13
	311	Northern	3.69
	312	South Coast	1.20
	313	South West	6.58
	314	Sunshine Coast	2.36
	315	West Moreton	3.06
	316	Wide Bay	4.30
South Australia	401	Eyre Peninsula	6.07
	402	Hills, Mallee, and Southern	3.55
	403	Metropolitan East	1.00
	404	Metropolitan North	1.06
	405	Metropolitan South	1.35
	406	Metropolitan West	1.00
	407	Mid North	5.12
	408	Riverland	5.07
	409	South East	5.00
	410	Flinders and Far North	6.15
	411	Yorke, Lower North, and Barossa	4.79
Western Australia	501	Goldfields	6.45
	502	Great Southern	5.22
	503	Kimberley	6.88
	504	Metropolitan East	1.34
	505	Metropolitan North	1.21
	506	Metropolitan South East	1.29
	507	Metropolitan South West	1.63
	508	Mid West	6.06
	509	Pilbara	6.81
	510	South West	4.00
	511	Wheatbelt	5.44
	512	Indian Ocean Territories	7.00
Tasmania	601	North Western	4.75
	602	Northern	4.21
	603	Southern	3.63
Northern Territory	701	Alice Springs	6.33
	702	Barkly	7.00
	703	Darwin	3.19
	704	East Arnhem	6.33
	705	Katherine	6.29
Australian Capital Territory	801	ACT	1.00

## Appendix C. Selected Occupations

There is no universally accepted definition of the aged care workforce or consensus on which occupations it includes.

The Aged Care Provider Workforce Survey, administered by the Department of Health and Aged Care, categorises occupations into allied health, nursing, and ancillary or support staff [33]. This analysis includes all occupation categories captured by the survey but widens it to include doctors, dentists, and community support workers to reflect the broader health workforce older adults rely on.

This analysis uses two data collection instruments to estimate the size and composition of the aged care workforce. Direct care workers—including doctors, nurses, allied health professionals and assistants, and personal support workers—were identified in the Census using the Australian Bureau of Statistics’ (ABS) ANZSCO—Australian and New Zealand Standard Classification of Occupations [34]. These codes aggregate occupations based on similar skills, tasks, and roles.

As the analysis focuses on aged care service delivery in community settings, not critical or acute care, efforts were made to exclude occupations limited to hospital settings. In addition, where the designation was unclear or its inclusion could arguably create analytical problems, these codes were excluded (see below for the treatment of nfd and nec codes).

Occupations indirectly involved in aged care, such as managers, cleaners, and maintenance staff, were identified in the Census data using the ABS Industry of Employment (INDP) [35] filter for the Residential Aged Care Industry. This approach avoids artificially capturing occupations whose work may not specifically support aged care.

However, this decision significantly narrows the scope of indirectly involved workers, excluding those employed in in-home care, Multi-Purpose Services (MPSs), and the National Aboriginal and Torres Strait Islander Flexible Aged Care Program (NATSIFAC).

The workforce delivering social and health support is complex and dynamic. While every effort was made to include the most relevant occupations related to aged care service delivery in Australia, while not including unnecessary codes that would artificially inflate it, some categorical overlap may persist, reflecting the inherent diversity of the aged care workforce.

### Appendix C.1. Groups, Their Definitions, and Occupation Code Inclusions

#### Appendix C.1.1. Allied Health Professionals and Assistants

There is no universally accepted definition of allied health. Rather, different governing bodies, governing departments, service providers, and insurers use slightly varying definition criteria and have a differing view of which health professions make up the allied health profession and workforce.

For ease, we are using a set of definitional criteria from the Allied Health Professions Australia (AHPA) [36], who define allied health professionals as follows:

Health professionals that are not part of medical, dental, or nursing professions.

University-qualified practitioners (qualified to bachelor’s or Australian Qualifications Framework 7 [37] or higher) with specialist expertise in preventing, diagnosing, and treating a range of conditions.

Governed by a national professional organisation with a code of ethics/conduct, clearly defined membership requirements, and robust and enforceable regulatory mechanisms.

Practitioners in an evidence-based paradigm, using an internationally recognised body of knowledge and a clearly defined scope of practice.

The group includes a diverse range of health professionals that provide care in a range of settings including social workers, psychologists, occupational therapists, podiatrists, and pharmacists. Allied health assistants, when trained and directly involved with the delivery of allied health, were also included even though they may not necessarily meet all the criteria set out by AHPA’s definition. For example, pathology collectors are instrumental in the delivery of pathology services but may need a skill level of AQF (Australian Qualifications Framework) 2 and 3, as opposed to a pathologist with an AQF-7.

Other occupations included in allied health and assistants that may not necessarily be qualified to QSF level 7 include counsellors who may qualify with an advanced diploma, associate degree, or bachelor’s or above (AQF-5 and above). Nonetheless, counsellors are considered to be allied health professionals by AHPA and are represented by a peak body that is a member of AHPA itself.

Conversely, complementary health professions were excluded even though some occupation in this category do meet some of the general principles for allied health care. Traditional Chinese Medicine (TCM) and naturopathy qualification can be (but are not required to be) attained in Australia at AQF level 7 (bachelor’s degree), and the TCM profession is regulated by Australian Health Practitioner Regulation Agency (AHPRA) [38], the body that also regulates all allied health professions nationally. TCM practitioners are also included in the national healthcare workforce dataset (NHWDS) [39], and many complementary therapies are covered under allied health by private health insurers. However, neither AHPA nor any state-based allied health governing body recognise TCM as an allied health profession (the ACT was the only state or territory to recognise TCM as allied health as of this article’s writing [40,41,42,43,44,45]), and it fails other key definitional criteria for allied health professionals. Thus, all complementary health therapist occupations (naturopaths, traditional Chinese medicine practitioners, homeopaths, and acupuncturists) are excluded from the allied health group, and from this analysis in general.

While dentists are uniformly excluded from allied health definitions, oral health professionals such as dental hygienists and therapist are included according to some governing bodies, including the Victorian and South Australian Departments of Health and the profession’s peak body (Dental Hygienists Association of Australia). However, to maximise the clarity and usefulness of this study, we have grouped oral health professionals with dentists (see below).

A decision was made to include health promotion officers in allied health as the minor label occupation group to which they belong (251 health diagnostic and promotion professionals) according to the Australian Bureau of Statistics uniformly and unambiguously includes only other allied health professions. Health promotion officers are often involved in the administration of the delivery of specialised allied health and diagnostic services, as opposed to general care management, although the role often calls for community-embedded care and education as well. In this way, health promotion officers can be thought of as assistants to the allied health workforce, although they often hold health qualifications at least to AQF-7 level.

#### Appendix C.1.2. Allied Health Professional and Assistant Occupation Categories

Exercise Physiologist
Nutrition Professionals nfd
Dietitian
Nutritionist
Medical Imaging Professionals nfd
Medical Diagnostic Radiographer
Medical Radiation Therapist
Nuclear Medicine Technologist
Sonographer
Optometrists and Orthoptists nfd
Optometrist
Orthoptist
Pharmacists nfd
Retail Pharmacist
Other Health Diagnostic and Promotion Professionals nfd
Health Promotion Officer
Orthotist or Prosthetist
Health Diagnostic and Promotion Professionals nec
Chiropractors and Osteopaths nfd
Chiropractor
Osteopath
Occupational Therapist
Physiotherapist
Podiatrist
Audiologists and Speech Pathologists/Therapists nfd
Audiologist
Speech Pathologist
Counsellors nfd
Drug and Alcohol Counsellor
Family and Marriage Counsellor
Rehabilitation Counsellor
Counsellors nec
Psychologists nfd
Clinical Psychologist
Psychotherapist
Psychologists nec
Social Worker
Medical Technicians nfd
Medical Laboratory Technician
Pharmacy Technician
Pathology Collector
Medical Technicians nec
Ambulance Officers and Paramedics nfd
Ambulance Officer
Intensive Care Ambulance Paramedic
Diversional Therapist

#### Appendix C.1.3. Personal Support and Care Workers

Personal support and care workers provide direct, personalised care and support to elderly Australians in a range of settings, including, but not limited to, the aged care services defined by the Department of Health and Aged Care.

Occupations such as nursing support workers, aged carers, and personal care workers provide essential daily care including social, emotional, communicative, therapeutic, or rehabilitative support.

This group also includes broader social support and welfare workers, including disability services officers, welfare workers, and Aboriginal and Torres Strait Islander Workers. These occupations often bridge personal care management and direct care provision and provide services such as assistance with planning and patient advocacy as well as direct patient health and welfare support.

All care types provided by this group are highly personalised and support older people to live more comfortable and integrated lives. They may provide assistance that supports better health and wellbeing outcomes, but these workers are not health professionals. Most occupations in this groups require a qualification of AQF level II or III, with the exception of a disability services officer, which may require AQF-5.

#### Appendix C.1.4. Personal Support and Care Worker Occupation Categories

Community Worker
Disabilities Services Officer
Residential Care Officer
Personal Carers and Assistants nfd
Aged or Disabled Carer
Nursing Support and Personal Care Workers nfd
Nursing Support Worker
Personal Care Assistant
Therapy Aide
Welfare Worker
Aboriginal and Torres Strait Islander Health Worker

#### Appendix C.1.5. Nurses

Nurses are trained to provide direct healthcare to patients in a range of clinical, residential, or home care settings. Registered nurses are qualified to bachelor’s degree or equivalent (AQF-7) and may specialise in particular areas of health care delivery. Enrolled nurses provide nursing care usually under the supervision of a registered nurse or other healthcare provider and are required to be qualified to AQF-5. This group includes nurse managers, nurse educators, and nurse practitioners, who are all highly qualified or specialised in an area of nursing area. While an attempt was made to exclude nursing occupations not directly related to community aged care provision (surgical and paediatrics), nurses who specialise in critical or emergency care were left in recognition that these occupations may work in residential aged care with nursing home units.

#### Appendix C.1.6. Nurse Occupation Categories

Nurse Educator
Nurse Manager
Nurse Practitioner
Registered Nurse (Aged Care)
Registered Nurse (Community Health)
Registered Nurse (Critical Care and Emergency)
Registered Nurse (Disability and Rehabilitation)
Registered Nurse (Medical)
Registered Nurse (Mental Health)
Registered Nurses nec
Enrolled Nurse

#### Appendix C.1.7. Doctors

Doctors have completed a medical degree and are trained to diagnose and treat a range of illnesses. While doctors work in a range of settings throughout the community and are intimately involved with the care of elderly people, an effort was made to exclude doctors and specialists with a focus clearly not to aged care delivery (for example, paediatricians and surgeons).

#### Appendix C.1.8. Doctor Occupation Categories

General Practitioners and Resident Medical Officers nfd
General Practitioner
Resident Medical Officer
Specialist Physician (General Medicine)
Cardiologist
Clinical Haematologist
Medical Oncologist
Endocrinologist
Gastroenterologist
Neurologist
Renal Medicine Specialist
Rheumatologist
Thoracic Medicine Specialist
Specialist Physicians nec
Psychiatrist
Otorhinolaryngologist
Urologist
Dermatologist
Obstetrician and Gynaecologist
Ophthalmologist
Pathologist
Diagnostic and Interventional Radiologist
Radiation Oncologist
Medical Practitioners nec

#### Appendix C.1.9. Dentists and Oral Health Professionals

Dentists and oral health professionals are a crucial part of any healthcare workforce. There is a difference between dentists, who are registered healthcare professions qualified with at least a four-year (AQF-7) degree to diagnose, trat, and manage dental diseases and injuries and generally maintain the dental health. Other oral health professionals such as hygienists and technicians are also qualified but to a lower level (usually AQF-5). Oral health professionals who are not dentists are often considered part of the broader allied health workforce, but we have included them here to ensure a cleaner, more practical analysis.

#### Appendix C.1.10. Dentists and Oral Health Professional Occupation Categories

Dental Practitioners nfd
Dental Specialist
Dentist
Dental Hygienists, Technicians, and Therapists nfd
Dental Hygienist
Dental Prosthetist
Dental Technician
Dental Therapist
Dental Assistant

#### Appendix C.1.11. Administration and Management

This group includes highly skilled occupations that are indirectly involved in the delivery or aged care, such as practice managers and retirement village managers. While these workers are vital to the provision of aged care, they are not usually directly involved in the personal care of older adults.

#### Appendix C.1.12. Administration and Management Occupation Categories

Health and Welfare Services Managers nfd
Medical Administrator
Nursing Clinical Director
Primary Health Organisation Manager
Welfare Centre Manager
Health and Welfare Services Managers nec
Retirement Village Manager
Environmental Health Officer
Occupational Health and Safety Adviser
Practice Managers nfd
Health Practice Manager
Practice Managers nec
Medical Receptionist
Recreation Officer
Occupational and Environmental Health Professionals nfd

#### Appendix C.1.13. Notes on Handling Nfd and Nec Codes

Occupation codes with labels ending in nfd (not further defined) are used when the response cannot be coded to a specific occupation but can be coded to a higher level (four-digit unit code or higher). These are excluded except in cases when every other six-digit category within the higher-level code was included to avoid including erroneous occupations.

Occupation categories with nec (not elsewhere classified) are similar to nfd but are less ambiguous because the designation remains in the smallest, most disaggregated group, rather than defaulting to a higher, broader category. In addition, it is possible to examine the occupations that make up nec codes. For this reason, where appropriate, nec codes have been included, even when not all six-digit codes were included in that group. An example is “Registered Nurses nec (254499)”, which includes the following specialisations: Aboriginal Health Nurse, Flight Nurse, Immunisation Nurse, Nursing Officer (Defence Forces), Pathology Nurse, Radiology Nurse, Red Cross Blood Service Nurse, Registered Nurse (Infection Control), and Remote Area Nurse. We reasoned that this classification code was relevant to the care of elderly people, even when other registered nurse categories (for example, paediatric nurses) were excluded.

## Appendix D. Age Distributions in Rural and Remote ACPRs

Figure A1 and Figure A2 show the age distributions of rural and remote regions, compared to metropolitan ACPRs. The figure shows considerable variability in the age profile across individual regions.

## Appendix E. Analysis of Care Need

Individuals in the Census aged 65 and over were allocated to four care categories, based on their need for assistance with core activities (ASSNP) and their number of long-term health conditions (CLTHP). In addition to the 65 and over group, a small number of younger residents of care facilities were included as these individuals require care that is drawn from the same workforce. Table A3 compares the distribution of care needs for the 71,665 individuals in residential care (the identification is based on code RNLP, the individual being “usually resident” in a “non-private dwelling” as a “guest, patient, inmate or other resident” for those aged 65 and over. Our previous article highlighted the relative under-provision of residential care facilities in rural and remote Australia) in rural ACPRs and 6674 in remote ACPRs, with 130,553 in metropolitan ACPRs.

**Table A3:** Australians aged 65 years and over in residential care.

	Aged-Care-Planning Region Type						
Care Needs	Metropolitan		Rural		Remote		All Residents
	*n*	% *	*n*	% *	*n*	% *	%
Highest care: assistance and 3 or more conditions, 65 years and over	29,973	23.0%	18,381	25.6%	1885	28.2%	24.1%
High care: assistance and 2 or less conditions, 65 years and over	59,255	45.4%	32,376	45.2%	2847	42.7%	45.2%
Medium care: long-term conditions but no assistance, 65 years and over	19,074	14.6%	9803	13.7%	828	12.4%	14.2%
Low care: no long-term conditions, 65 years and over	15,539	11.9%	7432	10.4%	813	12.2%	11.4%
Aged 20–64, inmates, need assistance	6712	5.1%	3673	5.1%	301	4.5%	5.1%
Total in residential care	130,553		71,665		6674		

* As a percentage of those in residential care.

Table A4 covers older adults living in their own homes. The first three rows show those requiring assistance with core activities or having complex health needs. These were grouped, using the need for assistance and number of long-term health conditions.

**Table A4:** Australians aged 65 years and over with in-home care needs.

	Aged-Care-Planning Region Type						
Care Needs	Metropolitan		Rural		Remote		All Residents
	*n*	% *	*n*	% *	*n*	% *	%
Requires highest care: assistance and 3 or more conditions	115,937	3.7%	66,139	3.8%	5579	3.3%	3.7%
Requires high care: assistance and 1 or 2 long-term conditions	209,641	6.7%	93,600	5.4%	8266	4.9%	6.2%
Requires medium care: assistance, no long-term conditions and no assist, 3 or more long-term conditions	221,015	7.1%	127,523	7.4%	11,909	7.1%	7.2%
No assistance, 2 or less long-term conditions (residual population 65 and over)	2,041,192	65.1%	1,155,872	66.8%	116,508	69.3%	65.8%
Total at home	2,587,785		1,443,134		142,262		

* As a percentage of the total with in-home care needs.

## Appendix F. The Australian Care Workforce

Table A5 shows the number of individuals recorded by the 2021 Census as available to work in aged care. For most categories, the number of care workers per person aged 65 years and over is greater in metropolitan ACPRs compared to rural and remote regions. The table highlights a relative shortage of doctors and dentists in rural regions and doctors, dentists, and allied health workers in remote communities. Table A6 provides an estimate of the number of hours worked by each category of worker.

**Table A5:** Availability of formal aged care workers by ACPR type.

	Aged-Care-Planning Region Type						
	Metropolitan		Rural		Remote		Total
	*n*	Per 65 s and over	*n*	Per 65 s and over	*n*	Per 65 s and over	
Population aged 65 and over	2,711,626		1,511,126		148,635		4,371,387
Direct care workers							
Administration	110,702	0.041	43,816	0.0290	4281	0.0288	158,799
Doctors	66,371	0.024	19,529	0.0129	1482	0.0100	87,382
Dental	36,480	0.013	12,220	0.0081	966	0.0065	49,666
Nurses	161,205	0.059	78,339	0.0518	7902	0.0532	247,446
Allied health	186,754	0.069	70,111	0.0464	5946	0.0400	262,811
Personal carers	252,111	0.093	141,658	0.0937	14,287	0.0961	408,056
Indirect care workers							
Indirect working in residential aged care	46,326	0.017	20,828	0.0138	1616	0.0109	68,770
Total direct and indirect	859,949	0.317	386,501	0.2558	36,480	0.2454	1,282,930

**Table A6:** Total hours worked each week by care workers.

	Aged-Care-Planning Region Type						
	Metropolitan		Rural		Remote		Total
Population aged 65 and over	2,711,626		1,511,126		148,635		4,371,387
	Hours worked per week	Per 65 s and over	Hours worked per week	Per 65 s and over	Hours worked per week	Per 65 s and over	Hours worked per week
Direct care hours							
Administration	4,350,074	1.6042	1,735,018	1.1482	164,846	1.1091	6,249,938
Doctors	2,408,912	0.8884	654,500	0.4331	52,427	0.3527	3,115,839
Dental	973,058	0.3588	332,013	0.2197	25,825	0.1737	1,330,896
Nursing	5,065,509	1.8681	2,437,119	1.6128	260,877	1.7552	7,763,504
Allied health	5,914,056	2.1810	2,182,039	1.4440	168,147	1.1313	8,264,242
Personal care	7,113,767	2.6234	4,018,180	2.6591	421,534	2.8360	11,553,480
Indirect care hours							
Indirect workers in aged care	1,403,013	0.5174	619,984	0.4103	49,674	0.3342	2,072,671
Total direct and indirect	27,228,389	10.0414	11,978,852	7.9271	1,143,330	7.6922	40,350,571
